# Racial Discrimination as a Traumatic Bedrock of Healthcare Avoidance: A Pathway Through Healthcare Institutional Betrayal and Mistrust

**DOI:** 10.3390/healthcare13050486

**Published:** 2025-02-24

**Authors:** Pedram Rastegar, L. Cai, Jennifer Langhinrichsen-Rohling

**Affiliations:** Health Psychology Ph.D. Program, Department of Psychological Science, University of North Carolina Charlotte, Charlotte, NC 28223, USA; prastega@charlotte.edu (P.R.); lcai1@charlotte.edu (L.C.)

**Keywords:** healthcare avoidance, medical trauma, healthcare institutional betrayal, racial discrimination, medical mistrust

## Abstract

*Objectives:* Experiences of racial discrimination within the healthcare system are potentially traumatic events (PTEs) that have been associated with lowered perceived trust in healthcare providers, ongoing symptoms of PTSD and depression, and anticipated healthcare avoidance. Based on the BITTEN trauma impact model, we test a pathway such that greater past healthcare discrimination would be associated with anticipated future healthcare avoidance among BIPOC college students. We posited that this direct relationship would be sequentially mediated by healthcare institutional betrayal (HIB) during one’s worst healthcare event and subsequently reduced trust in healthcare. *Methods:* Our model was tested in a subsample of undergraduate students, all of whom self-identified with at least one minoritized racial or ethnic identity (*n* = 472). Participants reported on their past experiences with racial discrimination in healthcare. Each then chose and described their worst and/or most traumatic previous healthcare experience. Subsequently, they indicated if this experience included acts of HIB and/or led to medical mistrust. Finally, they reported on the degree to which they anticipated engaging in future healthcare avoidance. *Results:* Our model explained 31% of the variance in anticipated healthcare avoidance. As hypothesized via BITTEN, greater HIB during one’s worst or most traumatic healthcare experience and resulting mistrust in healthcare sequentially mediated the relationship between past experiences of healthcare racial discrimination and anticipated future healthcare avoidance. However, a direct relationship between racial discrimination in healthcare and anticipated healthcare avoidance was retained. *Conclusions:* Racial discrimination is a potentially traumatic experience associated with deleterious health outcomes. Current results suggest that healthcare discrimination may drive BIPOC college students’ future healthcare avoidance both directly and through experiencing increased healthcare institutional betrayal during one’s worst healthcare experience and resultant mistrust in healthcare. Due to the crucial role both discrimination and HIB experiences may play in healthcare outcomes, greater organizational adoption of anti-racist trauma-informed healthcare and the enactment of deliberate system-level repair strategies post discrimination and/or HIB is critical. Understanding the interplay of racial discrimination, HIB, and medical mistrust is also likely to help us address and repair system-level factors leading to anticipated healthcare avoidance behavior among BIPOC emerging adults.

## 1. Racial Discrimination as a Traumatic Bedrock of Healthcare Avoidance: A Pathway Through Healthcare Institutional Betrayal and Mistrust

Racial discrimination within the healthcare system has an ongoing influence on the quality of healthcare services for Black, Indigenous, and People of Color (BIPOC) [1,2]. Prenatal care [3], chronic illness outcomes [4], and emergency room outcomes [5] are only a few of the many health situations in which racial disparities have been documented. Racial discrimination has also been associated with ongoing trauma symptoms [6], leading scholars to consider racial discrimination as a potentially traumatic event, or PTE [7,8]. Consistent with this, experiences of racial discrimination are frequently accompanied by an increased risk of depressive [9], anxious, and dissociative symptoms [10], higher likelihood of PTSD, and overall pervasive and chronic reductions in economic, physical, and mental health [11]. A recent literature review of 28 studies found that racial discrimination was consistently shown to be a predictor of future traumatic symptoms for Black, Asian, and other BIPOC individuals, especially among veteran populations [12]. Additionally, the negative trauma-associated effects of racial discrimination have been shown to hold across age, gender, and/or education level and medical conditions [13,14,15,16,17]. Given the above, it should come as no surprise that racial discrimination has been associated with increased healthcare avoidance behavior [18,19], delayed medical screening [20] and increased mortality rates [21,22]. Since avoidance behavior is described as a core feature of traumatic disorders [23] and racial discrimination has been shown to be a potentially traumatic experience, discerning the connection between discrimination and avoidance could lead to a more comprehensive understanding of a significant issue within the healthcare system. Despite this, the potential mechanisms underlying how racial discrimination within one’s healthcare experiences influences avoidance behavior, particularly in relation to medical mistrust and other PTEs like healthcare institutional betrayal (HIB), have yet to be fully investigated. This is the primary purpose of the current study.

### 1.1. Anticipated Healthcare Avoidance as an Outcome to Prevent

Avoidance behavior is a common concern in the aftermath of a variety of traumatic or potentially traumatic events [23]. Healthcare avoidance behavior, defined as delaying or outright refraining from needed medical services [24], can lead to a host of negative outcomes, including greater severity of illness, higher mortality risk, and increased health disparities in populations with higher avoidance levels [25,26]. Given the logistical issues that can emerge when tracking patient behavior over time, along with difficulties in measuring lack of approach behavior, anticipated healthcare avoidance has become the primary proxy measure for healthcare avoidance behavior. External causes of anticipated healthcare avoidance can include discomfort with screening procedures, lack of available health services, and the significant financial burden associated with available services [25]. Additionally, past negative patient–provider interactions and anticipated negative treatment have also consistently been associated with healthcare avoidance across multiple demographic groups, including people classified as obese [27], racial and ethnic minorities [19,28,29], transgender and gender non-conforming individuals [30], and rural populations [31]. Systemic inequity plays a considerable role in how racial discrimination influences avoidance behavior. However, understanding the specific traumatic impact of discrimination as well as the mechanisms by which discrimination leads to avoidance, may help uncover additional potential intervention targets which may aid efforts to prevent problematic health-related avoidance behavior. This study investigate these connections using a trauma-informed, healthcare-provider-generated framework: the BITTEN healthcare trauma impact model [32].

### 1.2. The BITTEN Model

**Figure 1:**
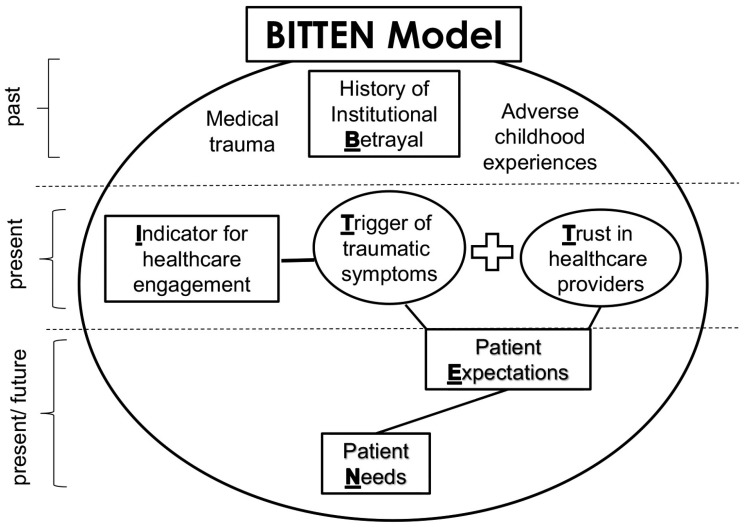
The BITTEN Trauma Impact Model depicts the impact of past traumatic experiences on present healthcare behaviors and perceptions of care [32].

**Figure 2:**
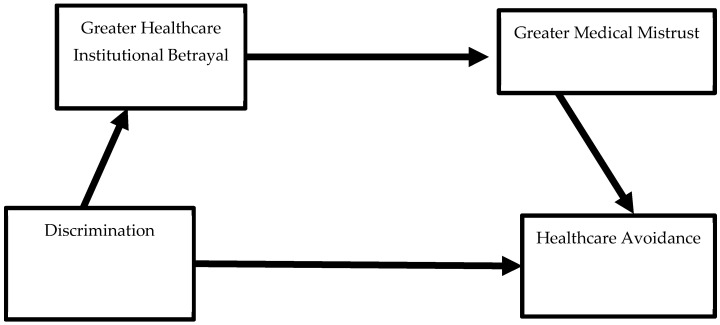
Conceptual Model of Healthcare-Related Racial Discrimination, a potentially traumatic event, predicting Anticipated Healthcare Avoidance sequentially through the greater experience of Healthcare Institutional Betrayal and Mistrust in Healthcare during and after BIPOC college students’ worst or most frightening previous healthcare experience.

Recently advanced by Lewis and Langhinrichsen-Rohling, the BITTEN trauma impact framework (**B**etrayal or trauma history, **I**ndicator of current problem, **T**rauma symptoms and **T**rust reduction activated in the current encounter, resulting in future **E**xpectation changes, and **N**eeds changes) describes the pathway by which traumatic past healthcare experiences can impact current healthcare encounters and generate future healthcare avoidance [32]. The authors posit that experiences of healthcare institutional betrayal, or HIB, can activate trauma symptoms during the current encounter, which can, in turn, negatively influence patient–provider trust, future care expectations, and needs in ways that can lead to anticipated healthcare avoidance [32]. In the BITTEN framework, past traumatic experiences are brought with the patient when they seek treatment for their presenting concern (i.e., the “indicator” in the framework, as seen in Figure 1). Additionally, depending on the characteristics of the indicator, activation of trauma history also increases the risk of additional healthcare institutional betrayal and increased mistrust, leading to a potential positive feedback loop of HIB sustained over time. While this model was initially generated to highlight the need for providers to assess for, prevent, and repair patients’ experiences of HIB, the framework notes that other PTEs, such as healthcare-related racial discrimination, could also be activated, thereby increasing the likelihood of medical mistrust and anticipated healthcare avoidance in a similar manner. To date, however, no published studies have considered how racial discrimination might function as an initial activating trauma or PTE in the BITTEN model. Specifically, no existing literature has considered how this pervasive and systemic type of potentially traumatic experience could be related to healthcare avoidance through the experiences of greater healthcare institutional betrayal and reduced healthcare trust in a problematic healthcare encounter (See Figure 2). This is the focus of the current study.

### 1.3. Healthcare Institutional Betrayal (HIB) as a Mediator

HIB is a type of betrayal trauma that occurs in the context of insufficiently protective or actively detrimental care provided by a healthcare system, including a hospital, health insurance company, an individual provider or representative within a system, or any other health-affiliated institution or service. Specifically, it is defined as an act (commission) or refusal to act (omission) perpetrated by a healthcare institution that should reasonably be expected to care for and protect the patients it serves [33]. HIB can include actions by the healthcare institution that run counter to best practice expectations (e.g., dismissing patient symptoms, gaslighting, careless misdiagnosis, lack of communication about cost or wait times) or negative systemic reactions to a patient reporting mistreatment or poor care (e.g., dismissing negative patient experiences or reports of injustice; poor response to whistleblowing). HIB experiences are distinct from medical trauma, in that broken trust between the institution (the healthcare system) and the patient is the primary source of distress; the act of betrayal by the system is considered damaging regardless of the severity of the medical event or even consciousness of the betrayal [33]. In contrast, medical trauma can include experiences of intense pain, fear, or poor outcomes, regardless of the healthcare system’s intention or behavior. In other words, the difference between HIB and medical trauma lies in the sources of the distress (betrayal of the trust relationship versus characteristics of the medical event). In line with understanding HIB as a betrayal trauma, experiences of HIB have been associated with higher risk for PTSD [34], particularly if accompanied by early maladaptive schema generated from other historically traumatic experiences such as adverse childhood events [24].

HIB may play a mediating role in the relationship between racial discrimination experienced in the healthcare setting and healthcare avoidance, as membership of an ethnic or racial minority group increases the likelihood of sustaining healthcare discrimination, which in turn increases the likelihood of betrayal. For instance, a prior study found that among a group of Black and Hispanic women and non-binary people, those who endorsed experiencing higher levels of racism also reported experiencing more institutional betrayal (IB) in the legal system [35]. Another study found that, among minoritized racial and ethnic survivors of sexual violence, 92% reported experiencing some form of institutional betrayal when reporting said violence to authorities [36]. Strong associations between experiences of discrimination and institutional betrayal have also been observed in general [34]. However, since experiencing HIB requires a patient to have inherent trust in the healthcare system, it is theoretically possible that the influence of HIB on anticipated healthcare avoidance may differ for populations who have historically experienced discrimination from the healthcare system, since historical and systemic racial discrimination is associated with lowered perceived trust in healthcare at the outset [7]. Unfortunately, little research has explored the relationship between racial discrimination and institutional betrayal within the healthcare system. As such, the role HIB plays in the healthcare avoidance behavior of BIPOC populations would benefit from further investigation.

### 1.4. Reduced Trust as a Mediator

In addition to HIB, trust may also play a mediating role in the relationship between discrimination and anticipated healthcare avoidance. Specifically, medical mistrust is another well-documented cause of healthcare avoidance behavior [37,38,39], especially for marginalized populations (e.g., BIPOC populations, LGBTQIA+, people of low SES). Multiple factors have been associated with increased mistrust levels, including previous negative healthcare experiences [39], poor insurance status [25], and anticipated or prior experiences with racial discrimination [40]. In addition, previous negative experiences with healthcare systems can significantly influence an individual’s “subsequent healthcare-seeking behaviors” [41]. As such, understanding the relationship between medical mistrust and healthcare avoidance behavior is also critical to reducing the negative public health impacts associated with avoidance of care.

It is important to note, however, that, to our knowledge, no research has examined the relationships among racial discrimination, HIB, and medical mistrust and anticipated healthcare avoidance simultaneously. Therefore, the mechanisms that explain how racial discrimination predicts healthcare avoidance are unclear. Given that past research has indicated that discrimination is broadly related to experiences of IB [35] and that both discrimination and HIB contribute to higher endorsement of medical mistrust [40,42], we posit that that discrimination will predict higher levels of healthcare avoidance through both HIB and medical mistrust. Consequently, we hypothesize a sequential mediating role such that discrimination in the healthcare setting will predict higher levels of HIB which in turn will predict greater levels of medical mistrust and ultimately will lead to higher healthcare avoidance. Understanding the direct and indirect pathways to healthcare avoidance is a necessary next step.

In summary, the main aim of this study is to examine the proposed relationships between racial discrimination, HIB, subsequent medical mistrust occurring in a worst or most frightening healthcare event, and overall healthcare avoidance behavior within a trauma-centered framework for healthcare experiences (i.e., BITTEN). Based on the BITTEN model and previous literature regarding the impacts of other PTEs, we hypothesized that greater self-reported discrimination from the healthcare system would be directly associated with increased intention for healthcare avoidance among BIPOC college students. Furthermore, we expected that this relationship would be sequentially mediated by greater experiences of HIB during BIPOC participants’ self-reported worst past healthcare experience and their reports of decreased trust in healthcare in the aftermath of HIB. It is also anticipated that the addition of HIB and medical mistrust to the sequential model will increase the variance the model accounts for in comparison to a direct model from racial discrimination in healthcare to anticipated healthcare avoidance.

## 2. Methods

### Participants and Procedures

Data for this study were drawn from an existing database containing responses from 956 college student participants. Participants were originally recruited to describe the impact of their previous negative healthcare experiences on their COVID-19 pandemically related healthcare behavior. For this study, we focused exclusively on participants who self-identified as holding at least one minoritized racial or ethnic identity. All college student participants who self-identified with a BIPOC identity were retained for analysis, including individuals who reported White and another racial identity, as well as those identifying as White and reporting Hispanic/Latino for their ethnicity.

In total, 472 total BIPOC university student responses (Mage = 19.74 years, SD = 3.96) were included in this study, with ages falling between 18 and 62. Full demographic details of this sample can be found in Table 1. The participant population was diverse with many identifying with more than one racial identity: 17.8% White, 36.7% Black/African American/Afro-Caribbean, 13.6% South Asian, 6.7% multiracial, 10.6% East Asian, 5.3% Middle Eastern/Arab, 2.5% Native American, and 1.3% Pacific Islander. In terms of ethnic identity, 14.5% of the sample identified as Hispanic/Latino. This group constitutes the BIPOC sample (i.e., Black, Indigenous, People of Color). Lastly, regarding gender identity, 32% identified as men, 63% as women, and 4% as gender minority.

All participants were undergraduate students enrolled at a large public university located in a growing urban city in the mid-Atlantic and southeastern portion of the US. Each consented to complete a Qualtrics survey in partial fulfillment of a research requirement for an introductory psychology course; the studies were conducted as part of a SONA participant pool. IRB approval for this study was obtained and ethical procedures were followed throughout.

Participants who completed the survey in an inordinately quick fashion (less than 2 min), who completed 3% of the survey or less, or who either did not answer or incorrectly answered the third attention check question which was embedded at the end of the survey were excluded from the analysis sample. Preliminary analysis of participant records determined that over 82% of the records that were completed in less than 2 min were mostly left blank (i.e., less than 3% of the information was in the record). Additionally, the few records that did have a higher percentage of information, even though the survey was finished exceptionally quickly (i.e., fewer than 5 records out of the 158) would have been disqualified due to other cleaning factors (e.g., failed attention checks). Thisinformation supported the 2-min or less cut-off decision for exclusion. 

Contents of the on-line survey included demographic items, a self-report measure of their experiences with racial discrimination when receiving healthcare, written responses describing participants’ worst healthcare experience, and then measures of the degree to which this worst experience contained acts of healthcare institutional betrayal, subsequent medical mistrust, and/or led to anticipated healthcare avoidance. Participants were compensated with course credit following completion of the on-line survey. Other options to obtain course credit were readily available and participants were able to choose not to answer any of the question presented to them. Thus, the *n* varies slightly across analyses.

## 3. Measures

### 3.1. Demographics

Participants self-reported their race by choosing one of the following: White, Black/African American/Afro-Caribbean, American Indian/Alaskan Native/First Nations, East Asian/East Asian American, South Asian/South Asian American, Middle Eastern/Arab/North African, Native Hawaiian or other Pacific Islander, Identity not listed (please specify). Participants were allowed to select more than one option or choose multiracial. Regarding gender identity, participants self-reported their gender from a list of seven options with the opportunity to write in an option not listed. Participants also reported on their sexual orientation via a list of nine options that presented sexuality on a spectrum; they were also given the opportunity to self-describe an option not listed. Lastly, participants self-reported their current age and their year in school.

### 3.2. Racial Discrimination in the Healthcare Setting

Racial discrimination in the healthcare setting was measured through three items that were drawn from the Everyday Discrimination Scale [43] and Experiences of Discrimination within healthcare environments measure [44] which had been utilized previously [45,46]. A psychometric study recommended using either a single item or a multi-item measure to capture discrimination in the medical settings [46]. Due to time constraints associated with our multi-measure survey study, we chose to use the recommended single item along with two items drawn from the proposed multi-item measure. These three items asked participants to assess the extent to which they have: been discriminated against or made to feel inferior while receiving healthcare (the recommended single item); were treated with less respect or courtesy (two items from the multi-item measure were combined); or received poorer service than other people while receiving healthcare because of their race or ethnicity (item retained as written). We chose not to use items from the multi-item measure that asked about perceived discrimination from specific doctors and nurses as we were interested in the medical care system as a whole. Response options captured the frequency of occurrence on a scale from never (1) to four or more times (4). Items were summed to compute an overall racial discrimination in healthcare score, with higher values indicating more frequent experiences of healthcare discrimination. Our constructed 3-item measure had excellent internal consistency in the current study (α = 0.91).

### 3.3. Worst Healthcare Experience

Participants were presented with the following: “Now, think about your WORST/MOST FRIGHTENING healthcare experience when answering the following questions. Describe why you sought healthcare in the first place when you had your WORST healthcare experience”. Participants were then given a free response text box to answer why they sought healthcare during this experience. Participants were then told to continue to reflect on their worst healthcare experience as they assessed if this included acts of HIB, enhanced mistrust in their healthcare team, or led to anticipated healthcare avoidance through the use of the following measures.

### 3.4. Healthcare Institutional Betrayal

HIB was assessed using the Institutional Betrayal Questionnaire-Health (IBQ-H) [47] which is a 12-item dichotomous response act-based measure. Participants endorsed either “yes” or “no” to questions about if the healthcare system behaved in a variety of ways during their worst healthcare experience (e.g., did they mishandle your protected personal information, give you incorrect or inadequate information or advice that was not feasible for you to follow). Items were summed to compute an overall HIB score, with higher scores indicating higher endorsement of acts of HIB during one’s worst healthcare experience. The measure had good internal consistency in the current sample (α = 0.83).

### 3.5. Medical Mistrust

Medical mistrust was measured through an adaptation of the Wake Forest Physician Trust Scale [48]. Questions were modified to ask about trust in the entire healthcare team, rather than in a specific physician, after their worst healthcare experience. Item responses ranged from strongly agree (1) to strongly disagree (5) with higher scores indicating higher levels of mistrust. The measure had excellent internal consistency in this study (α = 0.91).

### 3.6. Anticipated Healthcare Avoidance (HAV)

Anticipated healthcare avoidance was measured via three items that were previously used to assess intention to avoid healthcare. These items had good properties in a study investigating the relationship between indicators of childhood trauma, HIB, and healthcare avoidance [24]. The items assessed the extent to which participants avoided or delayed seeking healthcare, were fearful of seeking healthcare, or had changed their approach to seeking healthcare (e.g., now they only go to the doctor if symptoms are severe) since their worst healthcare experience. Participants rated their frequency of each type of healthcare avoidance on a scale from not at all (1) to very much (4). The items were summed to compute an overall healthcare avoidance score, with higher values indicating greater levels of anticipated avoidance. The measure had good internal consistency in this study (α = 0.81).

## 4. Preliminary Analyses

All data were analyzed using SPSS Version 28. Descriptive statistics were computed to ensure measures were normally distributed and had acceptable variance. Pearson’s correlations were conducted to explore relationships among variables. Gender identity and sexual orientation were used as covariates in the analysis models to control for important identities that may also be associated with focal variables. Gender identity was categorized into three groups: woman, man, and gender minority due to low response rates on various gender minority categories. Sexual orientation was also dichotomized (0 = straight/heterosexual, 1 = sexual minority). Demographics for these variables are also depicted in Table 1.

## 5. Primary Analyses

To test our hypotheses, we utilized Process Macro Model 6 in SPSS Version 28 which utilizes linear regression modeling to determine if there is an indirect effect of discrimination on healthcare avoidance through HIB and medical mistrust. Directionality of our analysis was based on theory. Bootstrapping was used to compute confidence intervals. Gender and sexual minority were used as covariates with women and straight/heterosexual as the reference groups. To detect mediation, we first explored a direct relationship between racial discrimination in healthcare, our covariates, and healthcare institutional betrayal (HIB). Next, we determined if racial discrimination in healthcare and HIB predicted medical mistrust. Finally, we tested if racial discrimination within the healthcare system, HIB, and medical mistrust all predicted anticipated healthcare avoidance. We specifically considered if there was an indirect relationship through both of our two proposed mediators. Serial mediation was detected if the confidence interval of the indirect effect of racial discrimination in healthcare, through both HIB and medical mistrust, did not include zero [49]. Descriptives for our primary measures are shown in Table 2.

## 6. Results

### 6.1. Associations Among Racial Discrimination, HIB, Medical Mistrust, and Anticipated Healthcare Avoidance

As expected and shown in Table 3, anticipated healthcare avoidance was significantly correlated with all focal variables including higher levels of experienced racial discrimination in healthcare (*r* = 0.32, *p* < 0.001), greater HIB (*r* = 0.48, *p* < 0.001), and medical mistrust (*r* = 0.46, *p* < 0.001) with small to moderate effects. Furthermore, racial discrimination in healthcare was positively associated with greater reports of HIB during one’s worst and most traumatic healthcare experience (*r* = 0.40, *p* < 0.001) and medical mistrust (*r* = 0.33, *p* < 0.001). HIB was moderately correlated with higher levels of medical mistrust (*r* = 0.59, *p* < 0.001). Lastly, sexual minority status was associated with higher reported levels of HIB, medical mistrust, and anticipated healthcare avoidance (*r* = 0.13 to 0.20, *p* < 0.01). The strength of these relationships was small.

### 6.2. Testing the BITTEN Model to Predict Anticipated Healthcare Avoidance

Full details of the results are reported in Table 4. First, our model predicting HIB was significant (*R*^2^ = 0.18, *p* = 0.000) and explained 18% of the variance. Greater healthcare-related racial discrimination predicted higher levels of HIB (β = 0.38, *p* = 0.000). Participants who identified as a sexual minority also reported higher levels of HIB (β = 0.17, *p* = 0.000). Gender identity was not associated with HIB.

Next, our model predicting medical mistrust was significant (*R*^2^ = 0.38, *p* = 0.000), explaining 38% of the medical mistrust variance. Experiences of racial discrimination in healthcare (β = 0.13, *p* = 0.002) and HIB during one’s worst or most frightening healthcare experience (β = 0.53, *p* = 0.000) both significantly predicted higher levels of medical mistrust. Neither sexual minority nor gender identity was associated with medical mistrust.

Finally, our full model predicting anticipated healthcare avoidance was significant (*R*^2^ = 0.31, *p* = 0.000) and explained 31% of the variance. Racial discrimination within healthcare (β = 0.11, *p* = 0.011), HIB (β = 0.28, *p* = 0.000), and medical mistrust (β = 0.26, *p* = 0.000) all predicted higher levels of anticipated healthcare avoidance. While men’s reports of healthcare avoidance did not significantly differ from women’s, participants who identified as a gender minority reported significantly higher levels of healthcare avoidance (β = 0.11 *p* = 0.008). Sexual identity did not predict healthcare avoidance.

Lastly, as predicted via the BITTEN model, there was a significant indirect effect of racial discrimination in healthcare (β = 0.05) on anticipated healthcare avoidance through HIB and medical mistrust based on the obtained non-zero confidence intervals. These results indicate partial mediation as the effects of racial discrimination contributed to anticipated healthcare avoidance both directly and indirectly through HIB and medical mistrust.

## 7. Discussion

Experiences of racial discrimination constitute potentially traumatic events (PTEs) that have been associated with known trauma symptoms [12] and deleterious health-related outcomes including avoidance [29]. Relatedly, experiencing racism has also been related to greater experiences of institutional betrayal in the criminal justice system [35]. Understanding health-related outcomes associated with this kind of PTE is an important health directive. The current study extends the healthcare trauma literature by focusing on racial discrimination that has occurred within the healthcare system, with the hypothesis that these experiences will leave BIPOC college students at risk for anticipated healthcare avoidance. This study also advances the field through the use of the BITTEN trauma impact framework, which describes how historical experiences of trauma, both from within and outside of the healthcare system, can affect both the current patient–provider encounter as well as patients’ future needs and expectations for care within the healthcare system.

One outcome that is important to predict is healthcare avoidance, as it has been associated with multiple deleterious effects including severity of illness and increased mortality [26]. Health-related prevention efforts generally require engagement (i.e., annual physicals, routine dental care); these efforts are likely to be weakened in the face of anticipated healthcare avoidance. In the current study, racial discrimination experiences that occurred while seeking past healthcare functioned as the bedrock for anticipated healthcare avoidance among BIPOC college students, maintaining a direct effect even in analyses that contained significant mediating variables. These findings suggest that continued health disparities can be expected if we cannot eradicate BIPOC individuals’ experiences of racial discrimination from the healthcare system. The need for anti-racist healthcare is supported. These findings also support the growing literature that recognizes racial discrimination as a potentially traumatic event.

This study was theoretically grounded in the BITTEN trauma-informed model of healthcare delivery. According to BITTEN, past experiences of trauma, such as those potentially associated with experiences of healthcare racial discrimination, function as a risk factor for healthcare institutional betrayal and medical mistrust in current patient–provider interactions [32]. Once activated in a problematic patient encounter, these three variables can then be expected to predict anticipated healthcare avoidance in BIPOC college students. Importantly, this model was supported in the current study, accounting for 31% of the variance in anticipated healthcare avoidance. Furthermore, both proposed mediators were retained as significant predictors in the final model.

There are multiple implications to these findings. First, there is a continued need to conceptualize patient interactions with systems of care through the lens of institutional betrayal, which in turn is rooted in betrayal trauma theory [50]. Betrayal trauma theory, drawn from sexual assault survivors’ experiences of secondary trauma at the hands of the criminal justice system, postulates that there is inherent betrayal when a patient relies on a healthcare institution to provide care and that implicit trust bond is broken, either through direct action (commission of harm) or through unexpected inaction (omission of care) [50]. Theoretically, however, betrayal trauma theory has been silent about how systemic harm or betrayal might occur in those less likely to imbue the healthcare system with implicit trust, particularly patients with a minoritized identity and those with a greater likelihood of experiencing past discrimination within the healthcare system. Thus, further elucidation on the impacts associated with institutional betrayal compared to other forms of betrayal trauma is needed.

Nonetheless, findings from this study fill a gap by utilizing the BITTEN trauma impact framework to conceptualize how discrimination experienced within healthcare can function as the bedrock of anticipated healthcare avoidance, both directly and through increasing the likelihood of experiencing healthcare institutional betrayal and resultant medical mistrust during one’s worst/most frightening previous healthcare experience. This study adds to the growing body of literature highlighting the health toll enacted by systemic racism within the healthcare system [13,14,15,16,17].

Furthermore, there are important implications that can be drawn from this study. First, given the deleterious effects of discrimination, federal as well as organizational policies to eliminate racist behaviors overall and within healthcare settings are needed. Additionally, healthcare settings can learn from the BITTEN model and utilize a patient-centered and trauma-informed care approach to healthcare. Healthcare providers can implement trauma-informed care by realizing the rate of trauma, recognizing the impact of trauma symptoms, and avoiding retraumatizing the patient [24,42,51]. Healthcare providers should assess for experiences of discrimination within the healthcare setting as well as past experiences of HIB. Awareness and understanding these experiences will allow the providers to begin repairing breaches of trust which can increase positive expectations for future healthcare [52] and ultimately reduce healthcare avoidance.

Limitations to this study should be noted. First, all data were collected via self-report which is subject to memory errors and shared method variance. Second, the design was cross-sectional, precluding a true understanding of the causal relationships among these variables as well as a clear picture of how these experiences unfold across time. Additionally, more refined measures of discrimination in the healthcare setting are needed. Other unmeasured variables, such as the level of actual avoidance compared to anticipated avoidance and the nature of the primary presenting concerns of people seeking healthcare during their worst experience, may also have important impacts on these relationships. Fortunately, the included variables in this study were grounded in theory and were designed to form a timeline from historical experiences of racial discrimination in healthcare to a potential traumatic/worst healthcare experience that might include acts of healthcare institutional betrayal and resultant medical mistrust and anticipated or future healthcare avoidance. Nonetheless, longitudinal work in this area will be critical.

Additionally, “race” and “ethnicity” are sometimes used interchangeably in the literature [53,54]. However, recent scientific standards have called for treating race and ethnicity as separate constructs [55]. In keeping with these recommendations, our study participants separately reported their race and ethnicity; however, both constructs were utilized when selecting individuals for the analysis sample of BIPOC participants in order to increase power. Furthermore, the discrimination measure used in the current study asked for experiences based on either race or ethnicity; future studies will need to disentangle effects based on race versus those based on ethnicity. Additionally, our study explored the relationship between discrimination and healthcare avoidance across BIPOC identities whereas there are mostly likely within group differences. It is imperative for future studies to explore these differences. Similarly, while a strength of this study was the inclusion of gender identity and sexual orientation status as covariates in our model, a direct focus on the potentially traumatic healthcare experiences of these individuals, using the BITTEN model, is warranted. Finally, this work was performed utilizing college students as participants. While the strength of the predictive model was robust, and identifying early risk for a lifetime of healthcare avoidance is important, care should be taken if generalizing these findings to other populations or individuals located in other geographical areas.

There are many directions for advancement from this work. First, the literature focused on understanding healthcare-related trauma is nascent, even though theory in this area has advanced. Second, there is an ongoing need to consider the effects of intersectionality (e.g., veteran status, gender, age, disability, sexual orientation) on the association between racial discrimination and trauma symptoms and outcomes. Third, prevention and intervention efforts will need to explicate the impacts associated with various ongoing forms of healthcare-related racial discrimination. For example, provider bias is a form of social discrimination in which providers withhold certain treatment options from certain populations based on the belief that these populations will not adhere to treatment [56]. Different types of traumatic lived experiences are likely to have different impacts. Further investigation into other types of discrimination may also be warranted to determine whether this mediation relationship is unique to racial discrimination or potentially due to other co-occurring vulnerabilities. For example, recent IB research on the impact of moral injury after institutional betrayal following experiences of sexual and gender-related violence has found similar traumatic symptom outcomes (anxiety, depression) along with increased chronic pain, disconnection from others, and mistrust [57] for cisgender women and SGM populations—populations that have historically had a higher risk of experiencing discrimination and sexual harassment.

In conclusion, racial discrimination within healthcare has already taken place for some BIPOC college students. Our findings suggest that this discrimination may be associated with anticipated healthcare avoidance both directly and through increased institutional betrayal during a problematic healthcare experience and resultant mistrust in healthcare. Due to the crucial role HIB may play in discrimination outcomes, there is a need to reduce experiences of HIB and repair breaches of trust that have occurred during the frightening or problematic healthcare encounters of at-risk populations. A pathway forward may be to promote trauma-informed and patient-centered healthcare rather than systems-centered care or treatment as usual [58]. Further investigation into the role of institutional repair strategies [52] along with institutional willingness to seek truth and acknowledge wrongdoing, otherwise known as institutional courage [59], could provide more information on the long-term benefits of trauma-informed care and awareness within the healthcare system. Similar mitigation of institutional betrayal’s effect on trauma symptoms in sexual assault survivors has emerged, with institutional courage lowering the risk of trauma symptoms [60]. Finally, investigation into the role of institutional courage in the context of healthcare with other types of traumatic experiences would provide more information on the potential cross-cutting nature of HIB and institutional betrayal at large as an intervention target for anticipated healthcare avoidance across populations.

## Figures and Tables

**Table 1:** Demographic Characteristics of the Analysis Sample.

	N	%
	472	
Race *		
White	84	17.8
Black/AA/Afro-Caribbean	173	36.7
East Asian	50	10.6
South Asian	64	13.6
American Indian/Alaskan Native	12	2.5
Native Hawaiian/Pacific Islander	6	1.3
Middle Eastern/Arab/N. African	25	5.3
Multiracial	64	13.6
Identity not listed (other)	46	9.7
Prefer not to answer	18	3.8
**Ethnicity**		
Hispanic/Latino	139	29.4
Not Hispanic/Latino	322	68.2
Prefer not to answer	11	2.3
**Gender Identity**		
Woman	308	65.3
Man	145	30.7
Gender minority	16	3.4
Prefer not to say	3	0.6
**Sexual Orientation**		
Straight/Heterosexual	352	74.6
Sexual minority	108	22.9
Prefer not to answer	12	2.5
**Year in College**		
Freshmen	212	44.9
Sophomore	153	32.4
Junior	70	14.8
Senior	34	7.2
Graduate student	1	0.2
Other	2	0.4

*Note*. * Total adds up to higher N than sample as participants could choose more than one response.

**Table 2:** Means and Standard Deviations for Racial Discrimination in Healthcare, HIB, Mistrust, and Healthcare Avoidance Measures.

	*N*	Min–Max	*M* (*SD*)
Racial Discrimination in Healthcare	462	1.0–12.0	4.20 (2.18)
Healthcare Institutional Betrayal (HIB)	458	0.0–12.0	2.94 (2.90)
Medical Mistrust	469	6.0–50.0	28.64 (10.05)
Anticipated Healthcare Avoidance	463	2.0–15.0	7.56 (3.21)
Age in Years	455	18.0–62.0	19.74 (3.96)

*Note.* N indicates the number of participants who responded with the relevant information. N varies slightly across analyses due to missing data.

**Table 3:** Bivariate Correlations Among Discrimination, Healthcare Institutional Betrayal, Medical mistrust, Anticipated Healthcare Avoidance, and reported Sexual Minority Identity.

		1	2	3	4	5
1.	Healthcare Avoidance					
2.	Racial Discrimination	0.32 ***	-			
3.	Healthcare Inst. Betrayal	0.48 ***	0.40 ***	-		
4.	Medical Mistrust	0.46 ***	0.33 ***	0.59 ***	-	
5.	Sexual Minority Status	0.13 **	0.09	0.18 ***	0.20 ***	-

*Note*. ** *p* < 0.01, *** *p* < 0.001.

**Table 4:** Regression Coefficients, Standard Errors, and Model Summary Information for Racial Discrimination predicting Healthcare Avoidance via Serial Multiple Mediators.

	Step 1			Step 2			Final Model		
		M_1_ (HIB)		M_2_ (Mistrust)			Y (HAV)		
Antecedent	*b*	*SE*	*p*	*b*	*SE*	*p*	*b*	*SE*	*p*
X (Discrimination)	0.53	0.06	0.00	0.61	0.20	0.00	0.17	0.07	0.01
M_1_ (HIB)	—	—	—	1.86	0.15	0.00	0.33	0.06	0.00
M_2_ (Mistrust)	—	—	—	—	—	—	0.08	0.02	0.00
Men	−0.06	0.28	0.82	−1.48	0.87	0.09	−0.47	0.29	0.10
Gender Minority	−1.48	0.81	0.07	1.36	2.49	0.59	2.17	0.82	0.01
Sexual Minority	1.15	0.32	0.00	1.85	0.98	0.06	−0.18	0.33	0.56
Constant	0.53	0.31	0.09	20.56	0.97	0.00	3.87	0.46	0.00
	**R^2^ = 0.18**			**R^2^ = 0.38**			**R^2^ = 0.31**		
	F (4,432) = 23.96 *p* = 0.00			F (5,431) = 53.63 *p* = 0.00			F (6,430) = 32.79 *p* = 0.00		

*Note:* HIB = Healthcare Institutional Betrayal, Mistrust = Medical mistrust, HAV = Anticipated Healthcare Avoidance.

## Data Availability

The data presented in this study are available on request from the corresponding author due to privacy restrictions.

## References

[1] Mohottige D., Davenport C.A., Bhavsar N., Schappe T., Lyn M.J., Maxson P., Johnson F., Planey A.M., McElroy L.M., Wang V. (2023). Residential structural racism and prevalence of chronic health conditions. JAMA Netw. Open.

[2] Yearby R. (2018). Racial disparities in health status and access to healthcare: The continuation of inequality in the United States due to structural racism. Am. J. Econ. Sociol..

[3] Alhusen J.L., Bower K.M., Epstein E., Sharps P. (2016). Racial discrimination and adverse birth outcomes: An integrative review. J. Midwifery Women’s Health.

[4] Doshi R.P., Aseltine R.H., Sabina A.B., Graham G.N. (2017). Racial and ethnic disparities in preventable hospitalizations for chronic disease: Prevalence and risk factors. J. Racial Ethn. Health Disparities.

[5] Agarwal A.K., Gonzales R.E., Sagan C., Nijim S., Asch D.A., Merchant R.M., South E.C. (2024). Perspectives of Black patients on racism within emergency care. JAMA Health Forum.

[6] Pieterse A.L., Carter R.T., Evans S.A., Walter R.A. (2010). An exploratory examination of the associations among racial and ethnic discrimination, racial climate, and trauma-related symptoms in a college student population. J. of Couns. Psychol..

[7] Hall O.T., Jordan A., Teater J., Dixon-Shambley K., McKiever M.E., Baek M., Garcia S., Rood K.M., Fielin D.A. (2022). Experiences of racial discrimination in the medical setting and associations with medical mistrust and expectations of care among Black patients seeking addiction treatment. J. Subst. Abus. Treat..

[8] Carter R.T. (2007). Racism and psychological and emotional injury: Recognizing and assessing race-based traumatic stress. The Couns. Psychol..

[9] Polanco-Roman L., DeLapp R.C., Dackis M.N., Ebrahimi C.T., Mafnas K.S., Gabbay V., Pimentel S.S. (2022). Racial/ethnic discrimination and suicide-related risk in a treatment-seeking group of ethnoracially minoritized adolescents. Clin. Child Psychol. and Psychiatry.

[10] Polanco-Roman L., Danies A., Anglin D.M. (2016). Racial discrimination as race-based trauma, coping strategies, and dissociative symptoms among emerging adults. Psychol. Trauma: Theory Res. Pract. Policy.

[11] Cénat J.M. (2023). Complex racial trauma: Evidence, theory, assessment, and treatment. Perspect. Psychol. Sci. A J. Assoc. Psychol. Sci..

[12] Kirkinis K., Pieterse A.L., Martin C., Agiliga A., Brownell A. (2021). Racism, racial discrimination, and trauma: A systematic review of the social science literature. Ethn. Health.

[13] Bergeron G., Lundy De La Cruz N., Gould L.H., Liu S.Y., Levanon Seligson A. (2020). Association between racial discrimination and health-related quality of life and the impact of social relationships. Qual. Life Res. Int. J. Qual. Life Asp. Treat. Care Rehabil..

[14] Carter S.E., Ong M.L., Simons R.L., Gibbons F.X., Lei M.K., Beach S.R.H. (2019). The effect of early discrimination on accelerated aging among African Americans. Health Psychol. Off. J. Div. Health Psychol. Am. Psychol. Assoc..

[15] Cave L., Cooper M.N., Zubrick S.R., Shepherd C.C.J. (2020). Racial discrimination and child and adolescent health in longitudinal studies: A systematic review. Soc. Sci. Med..

[16] Thomas M.D., Michaels E.K., Reeves A.N., Okoye U., Price M.M., Hasson R.E., Chae D.H., Allen A.M. (2019). Differential associations between everyday versus institution-specific racial discrimination, self-reported health, and allostatic load among Black women: Implications for clinical assessment and epidemiologic studies. Ann. Epidemiol..

[17] Villarose L. (2022). Under the Skin: The Hidden Toll of Racism on American Lives and on the Health of Our Nation.

[18] Holder-Dixon A.R., Adams O.R., Cobb T.L., Goldberg A.J., Fikslin R.A., Reinka M.A., Gesselman A.N., Price D.M. (2022). Medical avoidance among marginalized groups: The impact of the COVID-19 pandemic. J. Behav. Med..

[19] Zhang D., Li G., Shi L., Martin E., Chen Z., Li J., Chen L., Li Y., Wen M., Chen B. (2022). Association between racial discrimination and delayed or forgone care amid the COVID-19 pandemic. Prev. Med..

[20] Powell W., Richmond J., Mohottige D., Yen I., Joslyn A., Corbie-Smith G. (2019). Medical mistrust, racism, and delays in preventive health screening among African-American men. Behav. Med..

[21] Farmer H.R., Wray L.A., Thomas J.R. (2019). Do race and everyday discrimination predict mortality risk? Evidence from the health and retirement study. Gerontol. Geriatr. Med..

[22] Cobb R.J., Sheehan C.M., Louie P., Erving C.L. (2022). Multiple reasons for perceived everyday discrimination and all-cause mortality risk among older black adults. J. Gerontol. Ser. A.

[23] American Psychiatric Association (2022). Trauma-and Stressor-Related Disorders. Diagnostic and Statistical Manual of Mental Disorders.

[24] Rastegar P.J., Langhinrichsen-Rohling J. (2024). Understanding college students’ healthcare avoidance: From early maladaptive schemas, through healthcare institutional betrayal and betrayal trauma appraisal of worst healthcare experiences. Healthcare.

[25] Kannan V.D., Veazie P.J. (2014). Predictors of avoiding medical care and reasons for avoidance behavior. Med. Care.

[26] Byrne S.K. (2008). Healthcare avoidance:A critical review. Holist. Nurs. Pract..

[27] McGuigan R.D., Wilkinson J.M. (2015). Obesity and healthcare avoidance: A systematic review. AIMS Public Health.

[28] D’Anna L.H., Hansen M., Mull B., Canjura C., Lee E., Sumstine S. (2018). Social discrimination and health care: A multidimensional framework of experiences among a low-income multiethnic sample. Soc. Work Public Health.

[29] Slaughter-Acey J.C., Sneed D., Parker L., Keith V.M., Lee N.L., Misra D.P. (2019). Skin tone matters: Racial microaggressions and delayed prenatal care. Am. J. Prev. Med..

[30] Kcomt L., Gorey K.M., Barrett B.J., McCabe S.E. (2020). Healthcare avoidance due to anticipated discrimination among transgender people: A call to create trans-affirmative environments. SSM-Popul. Health.

[31] Spleen A.M., Lengerich E.J., Camacho F.T., Vanderpool R.C. (2014). Health care avoidance among rural populations: Results from a nationally representative survey. J. Rural Health.

[32] Lewis C.L., Langhinrichsen-Rohling J., Selwyn C.N., Lathan E.C. (2019). Once BITTEN, twice shy: An applied trauma-informed healthcare model. Nurs. Sci. Q..

[33] Smith C.P., Freyd J.J. (2014). Institutional betrayal. Am. Psychol..

[34] Tamaian A., Anstey H., Kokokyi S., Klest B. (2024). The impact of discrimination and institutional Betrayal on Canadian University Students’ Mental Health. J. Trauma Dissociation.

[35] Porter E.F., Mendoza M.P., Deng M., Horwitz S.D., Soltys M.A., Hattery A.J. (2024). Institutional betrayal and race in the civil legal system: A latent class analysis with survivors of intimate partner violence. Race Justice.

[36] Gómez J.M. (2022). Gender, campus sexual violence, cultural betrayal, institutional betrayal, and institutional support in US ethnic minority college students: A descriptive study. Violence Against Women.

[37] Pellowski J.A., Price D.M., Allen A.M., Eaton L.A., Kalichman S.C. (2017). The differences between medical trust and mistrust and their respective influences on medication beliefs and ART adherence among African Americans living with HIV. Psychol. Health.

[38] Simon K.A., Driver R., Rathus T., Cole A., Kalinowski J., Watson R.J., Eaton L.A. (2024). HIV information avoidance, HIV stigma, and medical mistrust among Black sexual minority men in the Southern United States: Associations with HIV testing. AIDS Behav..

[39] Wang J.C., Dalke K.B., Nachnani R., Baratz A.B., Flatt J.D. (2023). Medical mistrust mediates the relationship between nonconsensual intersex surgery and healthcare avoidance among intersex adults. Ann. Behav. Med..

[40] Bazargan M., Cobb S., Assari S. (2021). Discrimination and medical mistrust in a racially and ethnically diverse sample of California adults. Ann. Fam. Med..

[41] Schwei R.J., Johnson T., Matthews A.K., Jacobs E.A. (2017). Perceptions of negative health care experiences and self-reported health behavior change in three racial and ethnic groups. Ethn. Health.

[42] Selwyn C.N., Lathan E.C., Richie F., Gigler M.E., Langhinrichsen-Rohling J. (2021). Bitten by the system that cared for them: Towards a trauma-informed understanding of patients’ healthcare engagement. J. Trauma Dissociation.

[43] Williams D.R., Yu Y., Jackson J.S., Anderson N.B. (1997). Racial differences in physical and mental health: Socio-economic status, stress, and discrimination. J. Health Psychol..

[44] Krieger N., Smith K., Naishadham D., Hartman C., Barbeau E.M. (2005). Experiences of discrimination: Validity and reliability of a self-report measure for population health research on racism and health. Soc. Sci. Med..

[45] Bird S.T., Bogart L.M., Delahanty D.L. (2004). Health-related correlates of perceived discrimination in HIV care. AIDS Patient Care STDs.

[46] Hausmann L.R., Kressin N.R., Hanusa B.H., Ibrahim S.A. (2010). Perceived racial discrimination in health care and its association with patients’ healthcare experiences. Ethn. Dis..

[47] Smith C.P. (2017). First, do no harm: Institutional betrayal and trust in health care organizations. J. Multidiscip. Healthc..

[48] Hall M.A., Zheng B., Dugan E., Camacho F., Kidd K.E., Mishra A., Balkrishnan R. (2002). Measuring patients’ trust in their primary care providers. Med. Care Res. Rev..

[49] Hayes A. (2018). Process Macro for SPSS and SAS. Introduction to Mediation, Moderation, and Conditional Process Analysis.

[50] Birrell P.J., Freyd J.J. (2008). Betrayal trauma: Relational models of harm and healing. J. Trauma Pract..

[51] Selwyn C.N., Lathan E. (2021). Helping primary care patients heal holistically via trauma-informed care. J. Nurse Pract..

[52] Richie F.J. (2023). Repair Following Healthcare Institutional Betrayal. Ph.D. Dissertation.

[53] Benner A.D., Wang Y., Shen Y., Boyle A.E., Polk R., Cheng Y.-P. (2018). Racial/ethnic discrimination and well-being during adolescence: A meta-analytic review. Am. Psychol..

[54] Hackett R.A., Ronaldson A., Bhui K., Steptoe A., Jackson S.E. (2020). Racial discrimination and health: A prospective study of ethnic minorities in the United Kingdom. BMC Public Health.

[55] Flanagin A., Frey T., Christiansen S.L., AMA Manual of Style Committee (2021). Updated guidance on the reporting of race and ethnicity in medical and science journals. JAMA.

[56] Cipollina R., Sanchez D.T. (2023). Racial identity safety cues and healthcare provider expectations. Stigma Health.

[57] Kondrath S.R., Brandt E.A.B., Campbell K., Chamberlin E.S., Dordal P., East R., Fantus S., Frankfurt S.B., Golden K.B., Griffin B.J. (2024). Moral injury and institutional betrayal among cis women and sexual and gender minorities. Curr. Treat. Options Psychiatry.

[58] Campbell R. (2024). Systems-centered care versus survivor-centered care: Reimagining help and healing for sexual assault survivors. Psychol. Violence.

[59] Freyd J.J., Smidt A.M. (2019). So you want to address sexual harassment and assault in your organization? *Training* is not enough; *Education* is necessary. J. Trauma Dissociation.

[60] Adams-Clark A.A., Barnes M.L., Lind M.N., Smidt A., Freyd J.J. (2024). Institutional courage attenuates the association between institutional betrayal and trauma symptoms among campus sexual assault survivors. Psychol. Trauma Theory Res. Pract. Policy.

